# The Acacia (*Vachellia nilotica* (L.) P.J.H. Hurter & Mabb.): Traditional Uses and Recent Advances on Its Pharmacological Attributes and Potential Activities

**DOI:** 10.3390/nu16244278

**Published:** 2024-12-11

**Authors:** Lamiaa O. Hafez, Yeray Brito-Casillas, Noha Abdelmageed, Isabel M. Alemán-Cabrera, Samy A.F. Morad, Mahmoud H. Abdel-Raheem, Ana M. Wägner

**Affiliations:** 1 Department of Pharmacology, Faculty of Veterinary Medicine, Sohag University, Sohag 82524, Egypt; lamia.othman@vet.sohag.edu.eg (L.O.H.); nohafoud@googlemail.com (N.A.); 2 Instituto Universitario de Investigaciones Biomédicas y Sanitarias, Universidad de Las Palmas de Gran Canaria, 35016 Las Palmas de Gran Canaria, Spain; isabel.aleman@ulpgc.es; 3 Department of Pharmacology, Faculty of Veterinary Medicine, South Valley University, Qena 83523, Egypt; samy.morad@googlemail.com; 4 Department of Pharmacology, Faculty of Medicine, Assiut University, Assiut 71515, Egypt; ddr.mahmoudhamdy@yahoo.com; 5 Department of Endocrinology and Nutrition, Complejo Hospitalario Universitario Insular Materno-Infantil, 35016 Las Palmas de Gran Canaria, Spain

**Keywords:** *Acacia nilotica*, *Vachellia nilotica*, botany, ecology, traditional uses, phytochemistry, polyphenols, molecular docking, ethnopharmacological activities, toxicity

## Abstract

For thousands of years, *Vachellia nilotica* has been widely used as an herbal medicine to treat some diseases and symptoms, including respiratory, gastrointestinal and urogenital ailments. The present study was adapted to document and assemble existing information about *V. nilotica* and its evidence-based ethnopharmacological activities, with brief reviews on the description, geographical distribution, ecology, medical uses and phytochemistry. A literature review and information up to 2024 was performed in various scientific databases, including PubMed, Science Direct and Google Scholar. The keywords were “Acacia nilotica”, “Botany”, “ecology”, “Traditional uses”, “Phytochemistry”, “Polyphenols”, “Molecular docking”, “Ethnopharmacological activities” and “toxicity”, among others. *V. nilotica* has a wide range of uses, with low toxicity, reported in different countries. It can be infused into oils or tea or incorporated into paste, poultice and biscuits, used as an emollient, antidiarrheal, astringent and as an antidote for bite poisons. Glucose and lipid-lowering, anti-inflammatory, analgesic, antipyretic, antioxidant, antihypertensive, antibacterial, antifungal, antiviral and anthelmintic activities are the most prominent. Over 150 chemical components have been identified from *V. nilotica* that could be associated with its potential actions. Quercetin, rutin, kaempferol, naringenin, catechin, epicatechin, gallic acid, ellagic acid, lupeol and niloticane are its main active constituents. From the research data, and despite the fact that human clinical trials and detailed methodological studies are scarce, *V. nilotica* has shown wide-ranging activities, though the most robust evidence is related to the treatment of microbial infections, diarrhea, wound and ulcer healing and for topical application. More pharmacological and toxicological studies are required to further elucidate the mechanisms of action, potential side effects, and optimal dosages for these treatments. Additionally, more clinical trials are needed to validate these traditional uses in human populations and to ensure the safety and efficacy of *V. nilotica* for these applications. This article offers an overview of therapeutic applications by utilizing traditional uses and recent findings on phytochemical studies, and clinical and pharmacological research.

## 1. Introduction

**Figure 1:**
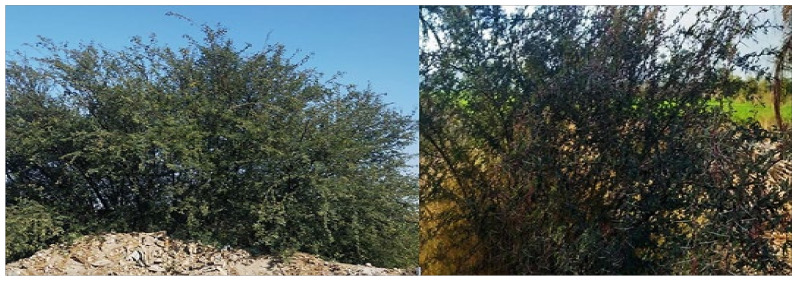
*Vachellia nilotica* (L.) Willd. ex Del tree at Sohag governorate in Egypt.

**Figure 2:**
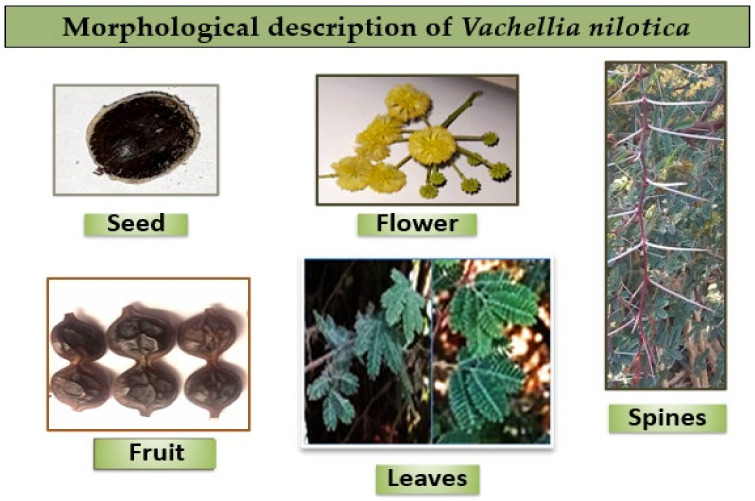
Morphological description of pods, seeds, flowers, leaves and spines of *Vachellia nilotica*.

*Vachellia nilotica* (L.) P.J.H. Hurter & Mabb., also known by the taxonomic synonym of *Acacia nilotica* (L.) Willd. ex Dellile (Figure 1 and Figure 2) is a leguminous tropical and subtropical tree belonging to the family *Fabaceae*. The genus name ‘Acacia’ was derived from the Greek word ‘akis’, meaning a point or a barb, and is related to the shape of its fruit’s pods. The species name nilotica refers to it being native to the Nile countries. The plant grows along the banks of canals crossing the Delta and the Valley of the Nile. It was grown in the past, though cultivation has stopped, and this species is occasionally seen along the canals near the Nile River. It can adapt to a wide range of climatic conditions. *V. nilotica* is one of the most important and frequently used traditional herbal medicines in tropical and subtropical regions. *V. nilotica* has long been utilized for leather tanning, biofuel generation, livestock feed and dyeing leather, wool, cotton and silk. The gum exudates from it used in foods, glues, inks and pharmaceutical preparations. It is valuable to agroforestry, traditional medicine and environmental sustainability [1,2,3,4,5,6,7,8,9].

*V. nilotica* has long been used to treat human and animal illnesses, it is valuable in both conventional and contemporary medicine and in more recent times, researchers have become interested in this practice. In addition, it is used as an emollient, antidiarrheal, astringent and antidote for bite poisons. Different medicinal benefits from several parts of the plant, including the pods, leaves, bark and flowers, have been demonstrated. *V. nilotica*’s medicinal properties are mainly attributed to its phytochemicals and bioactive components. The principal phytochemicals identified in this plant are tannins, flavonoids, alkaloids, saponins, glycosides, terpenoids, steroids, volatile oils and carbohydrates. Antibacterial, anti-inflammatory, antioxidant and anticancer properties are well-known to be exhibited by these substances [10].

Computational studies and in vitro/in vivo experiments have significantly increased our understanding of its medicinal properties and therapeutic potential. Computational approaches provide substantial insight into how bioactive compounds interact with possible protein targets [11]. These computational studies are supported by in vivo studies that have validated the efficacy of *V. nilotica* extracts in animal models, suggesting that they may be used to treat a variety of ailments. Numerous studies claim the therapeutic potential of *V. nilotica* in conditions like Alzheimer’s disease, hypertension, hyperlipidemia, inflammation, diabetes and cancer related to its active constituents. Illustrating the role of phytochemicals derived from *V. nilotica* and assembling in vitro/in vivo investigations are among the study’s objectives.

Studies on the possible medical potentials of *V. nilotica* pave the way for its incorporation into modern medical practices and could potentially lead to the development of new drugs that rely on the plant’s natural properties. This review aims to gather the latest findings on the pharmacodynamics, potential activities, toxicology, phytochemistry and overall attributes of *V. nilotica* and demonstrate the outstanding traditional and ethnomedicinal uses of this plant.

## 2. Search Strategy

This narrative review was conducted using articles retrieved from the following databases: PubMed, Science Direct and Google Scholar. The tailored terms used for these databases were combined using Boolean operators (AND and OR), incorporating these keywords: acacia, acacia nilotica, a. nilotica, vachellia, vachelia nilotica, v. nilotica, aplications, toxicology, traditional medicine, ethnopharmacology, ethnomedicine, ethnobotany, traditional use, composition, phytochemical, compounds, treatment, disease, diabetes, antioxidant, antifungal, antibiotic, antiparasitic, obesity, cardiovascular AND cancer. The relevant publications written in the English language from 1980 to 2024 were comprehensively reviewed. Original research conducted to evaluate *V. nilotica* pharmacological activities and previous reviews of the plant were evaluated and included in this article.

## 3. Taxonomical Hierarchy, Common Names, Ecology and Geographical Distribution

The taxonomical hierarchy and the common names of this plant are the following: kingdom: *Plantae*; subkingdom: *Tracheobionta*; phylum: *Spermatophyta*; subphylum: *Magnoliophyta*; class: *Magnoliopsida*; subclass: *Rosidae*; order: *Fabales*, family: *Fabaceae*, subfamily: *Faboideae*; genus: *Vachellia*; species: *Vachellia nilotica* [12].

The common names of *V. nilotica*, according to the different languages, are English (Egyptian thorn, Egyptian acacia, Nile acacia, Sunt wood, Prickly acacia, Black thorn, Scented thorn, Gum Arabic tree, Babul acacia, Thorny acacia, Thorn-mimosa); Spanish (Acacia gomífera); Arabic (Garad, sunt); French (Acacia à gomme, gommier rouge); Hindi (babul, Godi, Telia); German (Arabische Gummiakazie); Trade name (babul) [13].

Regarding its botanical description, *Vachellia nilotica* is a fast-growing tree that can reach heights of up to 15 m. It has a wide, rounded, or umbrella-shaped crown with low branches that are typically scattered, giving it a multi-stemmed appearance. The bark in young trees is green or slightly orange, while older trees have dark brown, rough bark that is furrowed and features deep, longitudinal fissures that exude gum. *V. nilotica* is spiny, and particularly in younger trees, the spines occur in pairs, are thin, sharp, either straight or deflexed and can reach lengths of up to 50 mm. The leaves consist of 3–10 pairs of pinnae. The roots are brown and deep, exhibiting lateral branching in older roots, while younger roots appear whitish. The fruits are oblong, indehiscent, leathery pods that range in color from dark brown to grey or grey-green and can be either straight or curved. Each pod contains 6 to 16 seeds, which are separated by constrictions, and the pods measure between 10 to 20 cm in length and 1 to 2 cm in width. The seeds are dark brown and subglobular. The flowers are bright yellow, sweetly scented, nectarless, globose heads and usually grow in clusters of 2 to 6 on pubescent stalks and are 1.2 cm in diameter (Figure 2). Most flowers are functionally male, with a few hermaphrodites and are mainly bee-pollinated [12,13,14].

**Figure 3:**
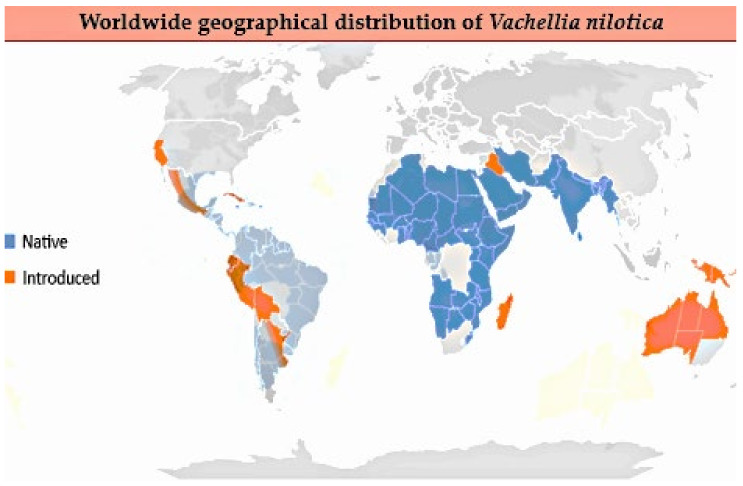
Worldwide geographical distribution of *Vachellia nilotica* updated according to Plants of the World Online on 24 August 2024 [16].

*V. nilotica* is a riverine nitrogen-fixing tropical and subtropical tree widely distributed in Africa, Asia, the Americas and Australia. Globally, *V. nilotica* is utilized in agroforestry systems as a source of lumber, fodder and green fertilizer trees. It has been discovered to significantly impact soil amendment, enhancing crop growth and yield performance [15]. Geographical distribution was recently updated according to Plants of the World Online [16] (Figure 3). *V. nilotica* thrives in average temperatures ranging from 15 to 28 °C and can withstand 50 °C but is sensitive to intense cold [17]. It grows in a variety of soils: moist, alluvial, saline, clay, poorer and well-drained soil [12].

## 4. Commercial Production Values

Tanning: *V. nilotica* has been traditionally used for the tanning of leather. The high tannin content in the pods and the cohesive molecular weight distribution of tannins provide good tanning properties through cross-linking capability with collagen by the formation of multiple hydrogen bonds. *V. nilotica* pods show high resistance to microbial activity and putrefaction [1,2] .

Dyes: Extracts from the bark, leaves and pods are used for dyeing leather, wool, cotton and silk. The dark brown color of *V. nilotica* and its dyeing properties are attributed to quercetin, acacetin and ellagitannins [3,4].

Gum: The gum exudate from vachellia trees varies from pale yellow to black depending on the amount of tannin it contains. It is soluble in water and has unique emulsification, film-forming and encapsulation properties. It is used in foods, baked goods and sweetmeats. In pharmaceuticals, it can be a carrier in capsules and in high soluble fiber supplements. It is also used in water colors, emulsion prints, glues and inks [5].

Timber: *V. nilotica* wood has easy mechanical and finishing properties, so it is suitable for furniture, wood decorations and shipbuilding [6].

Fuel: *V. nilotica* is used for the production of biofuel as a renewable energy source [12].

Food: The seed flour contains protein, fiber, fat, carbohydrates and microelements, such as potassium, magnesium, iron, phosphorus and manganese. The amino acids present in the seed flour are cysteine, methionine, threonine, lysine and tryptophan. Moreover, the seeds are considered a good source of minerals for bone formation evidenced by the Ca/P ratio of 1.20 [8]. In India, air-dried seeds are eaten when there is a scarcity of food resources and can also be used as food flavoring [9].

Fodders: *V. nilotica* can provide dry-season fodder for livestock, especially sheep and goats. Pods can be a source of nutritional energy and improve the efficiency of energy utilization in a concentrated mixture for ruminants [9].

## 5. Phytochemistry

**Figure 4:**
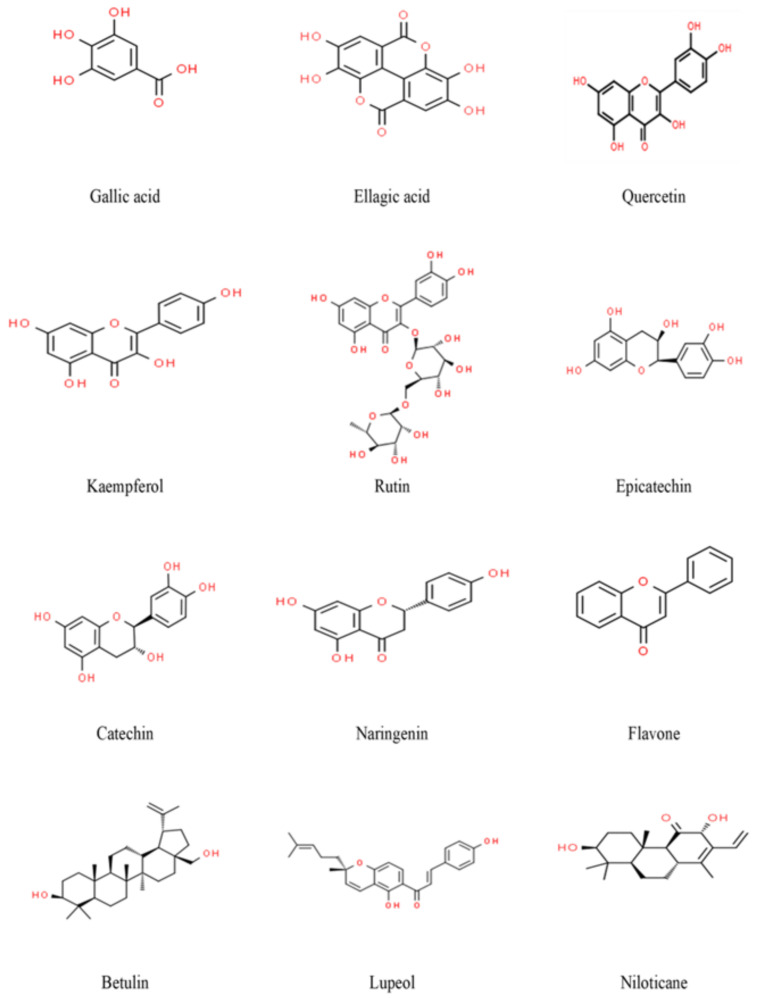
Structures of reported *Vachelia nilotica’s* phytochemical constituents (via ChemSpider, http://www.chemspider.com, accessed on 4 July 2023).

Over 150 chemical constituents have been identified from the genus vachellia [10]. *V. nilotica* possesses tannins, flavonoids, alkaloids, terpenes, saponins, proteins, polysaccharides and fatty acids. These active constituents have a variety of potential activity, such as anti-inflammatory, antioxidant, antipyretic, analgesic, antibacterial, antifungal, antiviral, glucose-lowering, lipid-lowering, anti-proliferative, antiulcer, antidiuretic and antidiarrheal activities. These potential activities are ascribed to its phytochemical constituents that actively interact with essential targets, exerting biological effects. The reported chemical constituents of *V. nilotica* are shown in Table 1 and Figure 4.

The target of the phytochemical constituents can be predicted by using computational techniques, such as virtual screening and molecular docking, frequently used in the discovery and development of new drugs. These approaches provide valuable insight into how bioactive compounds interact with potential protein targets, predicting the strength, stability and suitability of these interactions for drug development. By using these techniques, researchers can efficiently identify the most promising bioactive compounds from large libraries, accelerating the discovery of new therapeutic agents and refining potential mechanisms of action [11,32]. *V. nilotica*’s polyphenolic constituents, quercetin, rutin, kaempferol, naringenin, catechin, epicatechin, gallic acid and ellagic acid, proved to be those most likely responsible for its therapeutic activities. Table 2 illustrates some of these components and their predicted related mechanism of action as demonstrated through computational approaches, including molecular docking, molecular dynamics simulations, binding affinity predictions and ADMET analysis. Multiple computational tools supported these analyses. Molecular docking was predominantly performed using AutoDock Vina to evaluate the binding affinities between the phytochemicals and their potential protein targets. Pharmacokinetic properties and drug-likeness were assessed using Open Babel and Discovery Studio. In some studies, molecular dynamics simulations were conducted using iMODS and GROMACS to further refine the docking results and evaluate the stability of ligand-protein interactions. Sybyl and GOLD were also employed for molecular modeling and docking. The integration of these platforms allowed for detailed simulations throughout the studies.

## 6. Ethnomedicinal Uses

Using plant extracts in the therapy of human and animal diseases dates back to ancient traditions and has more recently triggered the interest of researchers [11]. *V. nilotica* can be infused into oils or tea or incorporated into paste, poultice and biscuits, used as an emollient, antidiarrheal, astringent and as an antidote for bite poisons. Table 3 illustrates the main ethnomedicinal uses and dosage forms of *V. nilotica*.

## 7. Pharmacodynamics and Potential Activities

*V. nilotica* has a wide range of pharmacological activities, such as glucose-lowering, antimicrobial, anti-inflammatory and anti-proliferative, based on in vivo and in vitro studies (Table 4). 

### 7.1. Glucose-Lowering Activity

The fruit leaves and bark of *V. nilotica* have shown improvements in blood glucose, plasma insulin, C-peptide, glycosylated hemoglobin (HbA1c), cholesterol, triglycerides (TG), high-density lipoprotein cholesterol (HDLC) and low-density lipoprotein cholesterol (LDLC) in different mouse, rat and rabbit models [32,64,67,68,69,70,71]. Oral administration of *V. nilotica* polyphenol leaf extract (250 mg/Kg and 500 mg/Kg) and glibenclamide (10 mg/Kg) for 4 weeks revealed significant activity in alloxan-induced diabetic rats by lowering the levels of the serum glucose and HbA1c and improving serum insulin and C-peptide levels compared with untreated diabetic rats regardless of their sex. Additionally, qRT-PCR analysis of the extract-treated rats’ pancreases demonstrated a strong regenerative effect on pancreatic beta cells through upregulation of the insulin signaling cascade, rat insulin gene (*Ins-1*), pancreatic and duodenal homeobox 1 (*Pdx-1*) (a regulator key for the transcription glucose-stimulated *Ins-1*, neurogenin 3 (*ngn3*) (a key gene for differentiation of pancreatic beta cells), insulin-regulated glucose transporter (*GLUT-4*), and insulin receptor substrate 1 (*IRS-1*). In addition, it downregulated the expressions of mitogen-activated protein kinase 8 (*MapK8*), tumor necrosis factor receptor-associated factor (*Traf-4* and *Traf-6*) genes, and reactive oxygen species (ROS) induced the c-Jun N-terminal kinase (JNK) signaling pathway, corroborative of the antioxidant defense activities [64]. Ethanolic leaf extract (200 mg/Kg/day, orally, for 20 days) displayed anti-hyperglycemic effects in alloxanized mice and improved both insulin resistance and cellular glucose uptake [73]. In vitro investigation of 70% ethanolic and aqueous pod extracts revealed highest α-glucosidase (carbohydrates hydrolyzing enzyme) inhibitory activity, with the half maximal inhibitory concentration (IC_50_) values 3.75  ±  0.62 μg/mL and 1.33  ±  0.57 μg/mL respectively, compared to acarbose as a positive control 240.00  ±  0.03 μg/mL (lower IC_50_ value corresponds to higher potency) [74]. Based on investigations, tannins may contribute to reducing postprandial hyperglycemia by inhibiting α-amylase and α-glucosidase. Increased insulin release from pancreatic β-cells has been associated with saponins found in *V. nilotica* [94].

### 7.2. Lipid-Lowering Activity

Dyslipidemia is defined as an elevation of cholesterol, TG, LDLC and/or lowering of HDLC levels that contribute to the development of atherosclerosis and cardiovascular diseases [95]. *V. nilotica* ethanolic leaf extract (30 mg/150 g/day, I.P., for 7 days and 4 weeks) in an adrenaline-induced hyperlipidemia rat (AIHRs) model showed a reduction of cholesterol, TG, LDLC and VLDLC (very low-density lipoprotein cholesterol), an increase in HDLC and a reduction in heart weight, left ventricular hypertrophy and cardiac myocyte size compared to untreated AIHRs [66]. These results were supported by another study in a fructose-induced hyperlipidemic rat model treated with *V. nilotica* pod ethanolic extract (200 mg/kg per day, for 7 days), which improved cholesterol, TG, LDLC and VLDLC and HDLC levels [69]. After the administration of the aqueous extract of *V. nilotica* leaves (300 mg/Kg/day, p.o., for 3 weeks) to streptozotocin (STZ)-induced diabetic rats, findings revealed a reduction in fasting blood glucose, TG and LDLC and an increase in serum insulin and HDLC compared with untreated diabetic rats. Thus, the authors suggested that *V. nilotica* might protect from atherosclerotic diabetic complications [65].

### 7.3. Antioxidant Activity

ROS plays a role in several disorders. Polyphenols and flavonoids present in *V. nilotica* act as ROS scavengers, diminishing lipid peroxidation generation and improving the antioxidant status. A *V. nilotica* polyphenolic leaf extract exhibited effective antioxidant activity in both in vivo and in vitro assays. In vivo studies in alloxan-induced diabetic rats treated with the polyphenolic extract (250 and 500 mg/Kg) and glibenclamide (10 mg/Kg) for 4 weeks showed significant inhibition in pancreatic and hepatic levels of ROS and thiobarbituric acid reactive substances (TBARSs) and elevation in glutathione peroxidase (GPx), superoxide dismutase (SOD), reduced glutathione (GSH) and catalase (CAT) levels compared with untreated diabetic rats [64]. Ethanolic leaf extract (10 μg/mL) showed potent antioxidant activity in vitro through 1,1-Diphenyl-2-picrylhydrazyl (DPPH) free radical scavenging assay in comparison with all the positive controls (ascorbic acid, tocopherol, quercetin and catechin) at the same dose, presumably due to the presence of considerable amounts of flavonoids and phenolic compounds [96]. The free radical scavenging activity of the ethanolic leaf extract was corroborated by two assays: total antioxidant activity and β-carotene bleaching assay [97]. Potent antioxidant activity of 70% ethanolic and aqueous pod extract was exhibited by the DPPH radical scavenging assay with an IC_50_ value of 4.06 ± 0.09 and 7.51 ± 0.19 μg/mL, respectively, versus the positive control Trolox (a derivative of vitamin E) 11.35 ± 0.05 μg/mL [74].

### 7.4. Anti-Inflammatory Activity

Anti-inflammatory activity of aqueous leaf extracts of *V. nilotica* at a dose of 150 mg/Kg body weight was determined in vivo by a formalin-induced inflammation test in Swiss albino mice, and results showed a 57.2% reduction in paw diameter, very similar to the response to diclofenac (dose not mentioned) as a reference drug which showed inflammatory inhibition by 56.3% [76]. Furthermore, the aqueous pod extracts at a dose of 100 mg/Kg reduced the carrageenan-induced rat paw edema to 64.4%, compared with 65.1% of the indomethacin (10 mg/Kg) as a positive control, and inhibited the granuloma formation induced by cotton pellets in rats to 25.6% in comparison with the reference drug dexamethasone (2.5 mg/kg) 37.6% [78]. Aqueous methanolic bark extract (50 µg/mL) inhibited tumor necrosis factor-α (TNF-α) stimulated 3T3-L1 adipocytes on the murine 3T3-L1 embryonic cell line by 50% compared with 29% inhibition by troglitazone (5 µg/mL) [30,68] reported that the niloticane (active constituents isolated from the bark of *V. nilotica* subsp. *Kraussiana)* has in vitro COX inhibitory effect with IC_50_ values of 28 μM against COX-1 and 210 μM against COX-2 compared with the values of indomethacin against COX-1 and COX-2, which are 3.6 and 189 μM, respectively. Betulin isolated from the bark of *V. nilotica* was found to be a COX-2 selective inhibitor assayed in vitro, resulting in inhibition of the COX-1 and COX-2 by 43.8% and 95%, respectively, at a concentration of 10 μM [28]. A randomized, placebo-controlled clinical trial was conducted to test the short-term clinical effects of a commercial gel containing *V. nilotica* in the treatment of plaque and gingival inflammation in patients with chronic generalized gingivitis. Results showed that vachellia gel has a significant reduction in gingival and plaque index scores compared to placebo gel, without teeth discoloration or unpleasant taste [61].

### 7.5. Neuroprotective Activity

Eldeen et al. (2010) also reported that niloticane has an in vitro cholinesterase inhibitory effect with an IC_50_ value of 4 μM compared with the value of 2.0 μM of the galantamine as a positive control. Inhibition of acetylcholinesterase improves neuronal transmission and may have potential in the treatment of neurocognitive disorders such as Alzheimer’s disease (AD) [30]. Furthermore, polyphenolics from *V. nilotica* pods have antioxidant activity and acetylcholinesterase inhibition effect against arsenic-induced neurotoxicity in mice [98]. The authors hypothesized that *V. nilotica* might have potential activities in the treatment of AD symptoms that are related to its cholinergic pathway besides their reported anti-inflammatory and antioxidant properties.

### 7.6. Analgesic and Antipyretic Activity

Analgesic effects of vachellia pod aqueous extract (500 mg/Kg) compared to aspirin (100 mg/Kg) as a positive control were estimated by the hot plate test on albino Swiss mice, which reflects a significant increase in reaction time compared to aspirin, reaching the maximum effect at 90 min after administration. In addition, it produced antipyretic activity, evaluated by yeast-induced pyrexia on Albino Wistar rats, albeit with less potency than aspirin [77]. Analgesic effects of vachellia bark aqueous extract for both acute and chronic pain were assessed in vivo by formalin-induced writhing assay in Swiss albino mice. The observations indicated both direct analgesic effects on the nociceptor blockage and inhibition of the synthesis and release of inflammatory pain mediators, though no mechanistic studies were described [76].

### 7.7. Antihypertensive and Antispasmodic Activity

The antihypertensive and antispasmodic properties of a *V. nilotica* methanolic pod extract were evidenced in vivo through lowering arterial blood pressure in rats in a dose-dependent manner (3–30 mg/Kg), both in systolic and diastolic blood pressure. In vitro studies showed an inhibition of the rate and force of spontaneous contractions in guinea-pig atria and rabbit jejunum, and the inhibition of serotonin-induced contractions in a dose-dependent fashion on isolated rat uterus. The antihypertensive and antispasmodic properties of vachellia were suggested to be related to its calcium and serotonin antagonistic action [92]. Ndamitso et al. (2017) elucidated that the mineral composition of seed flour has a Na/K ratio below one, indicating the potential effect of the flour as an antihypertensive agent by preserving body electrolyte balance [8].

### 7.8. Antiplatelet Activity

The methanolic extract of vachellia fruits inhibited the in vitro human platelet aggregation induced by the platelet-activating factors, adenosine diphosphate, arachidonic acid and collagen. This action was suggested to be due to Ca^2+^ channel blockade and a protein kinase C effect. In addition, it suppressed platelet aggregation mediated by the calcium ionophore A-23187, thus signifying the possibility of this effect through blockage of Ca^2+^ influx and also explaining its antihypertensive properties [93]. Significant inhibition of platelet aggregation (4.35%) was demonstrated in vivo in STZ-induced diabetic rats treated with 50 mg/Kg methanolic leaf extract for 3 weeks, compared to normoglycemic and untreated diabetic rats (2.11–8.76%), respectively [65].

### 7.9. Antibacterial and Antifungal Activity

Gupta and Gupta (2015) investigated the antibacterial efficacy of 50% *V. nilotica* as a mouthwash against salivary *Mutans streptococci* (*MS*) in high caries-risk human volunteers in a randomized controlled trial for 30 days followed by another 30 days without mouth wash. By culturing the collected saliva on mitis salivarius-bacitracin agar, the findings showed a significant decrease in the *MS* colony count in the *V. nilotica* and chlorhexidine groups (85% and 83%) at 30 days and (65% and 63%) at 60 days, respectively. These results reflected the similar antibacterial action of *V. nilotica* against *MS* to that of chlorhexidine [99]. Sadiq et al. (2017) evaluated the antibacterial activity of *V. nilotica* and elucidated its mode of action on foodborne and clinical strains of *Escherichia coli* (*E. coli*) and *Salmonella* spp. by observing changes in bacterial cell morphology and cell membrane integrity and permeability. Results showed substantial antimicrobial effects of vachellia against antibiotic-resistant bacterial strains [100]. The methanol and aqueous fruit cover extract at concentrations of 100%, 50%, 25% and 12% reflect antibacterial activity against gram-positive bacteria (*Staphylococcus aureus* and *Bacillus subtilis*) and gram-negative bacteria (*E. coli* and *Pseudomonas aeruginosa* by sensitivity test [101]. The effect of the vachellia bark decoction against bacterial vaginosis was tested in 45 patients in a single-blind, randomized, controlled clinical trial. The decoction was given orally (30 g twice daily) to 30 patients for one month and metronidazole (400 mg twice daily) to 15 women as control for 7 days. Results showed that *V. nilotica* has similar effects as metronidazole [88]. Leaf extracts were effective against *MS*, *Lactobacillus acidophilus*, *Fusobacterium nucleatum* and *Porphyromonas gingivalis.* Hence, it has the potential to be used as antiplaque and anticaries agents, as a herbal alternative to chlorhexidine [102,103,104]. Ali et al. (2018) evaluated the in vitro antibacterial and antifungal activities of bark petroleum ether and ethyl acetate extracts (300 µg/mL) against gram-positive bacteria (*Bacillus* and *Staphylococcus aureus*), gram-negative bacteria (*Salmonella*, *Shigella*, *Vibrio*, *Pseudomonas*, *Klebsiella* and *E. coli*) and fungi (*Candida albicans*, *Candida arrizae*, *Candida krusei*, *Aspergillus fumigatus*, *Aspergillus niger*, *Rhizopus oryzae* and *Saccharomyces cerevisiae*) by disc diffusion assay. The zone of bacterial inhibition for both extracts was around 9–13 mm, compared with Ciprofloxacin (10 µg/mL) 33–44 mm. The zone of fungal inhibition was around 7–8 mm compared with griseofulvin (25 µg/mL) 14–24 mm [75]. In vitro antibacterial and antifungal activities of the methanolic chloroform bark extract (75:25) through agar well diffusion assay against *Pseudomonas flurorescens*, *Bacillus subtilis*, *E. coli*, the zone of inhibition was 18, 22 and 8 mm, respectively, compared to Ciprofloxacin 22 mm, and against *Aspergillus niger*, was 6 mm compared with 20 mm of ketoconazole [79]. Aqueous, methanol, acetone and diethyl ether extracts of the bark and pods were highly effective in inhibiting the growth of *Penicillium italicum* and *Aspergillus niger*. In addition, effective reduction in mycelial weight and spore germination has been reported [105]. Ethyl acetate extract of *V. nilotica* seeds showed potent antifungal activity by inhibition of spore germination of *Candida albicans* (candidosis) and *Epidermophyton floccosum* (dermatophytosis) fungi [106]. Crude methanolic extract and its fractions demonstrated in vitro antibacterial activities against the oral bacteria *Streptococcus sobrinus* and *Porphyromonas gingivalis*, which are the main etiologic causes of dental caries [107]. Exacerbation of the microbial resistance problem and the need to control the use of antibiotics prompted the evaluation of plants as sources of potential chemotherapeutic and antimicrobial agents. *V. nilotica* could be an alternative antibacterial approach because of its safety, relatively low cost and effectiveness against multidrug-resistant pathogens [100].

### 7.10. Anti-Protozoan Activity

*Trypanosoma brucei* was cleared from infected mice circulation within 8 days of continued treatment by the crude methanolic stem extract (400 mg/Kg), while the partially purified extract (50 mg/Kg) cleared parasites from the circulation within 2 days [83]. The ethanolic leave extract of *V. nilotica* was assessed against *Giardia lamblia* trophozoites by in vitro susceptibility assays, and the findings revealed 100% inhibition by 500 µg/mL of the extract after 96 h compared with the standard drug metronidazole, which expressed 96% inhibition at concentration 312.5 µg/mL at the same time [84]. Bark ethanolic extract showed similar results against *Giardia lamblia* trophozoites [108]. In vitro investigation of methanolic extract of the fruits and bark against *Trichomonas vaginalis* exhibited potent 100% mortality at a concentration of 250 µg/mL, while chloroform bark extracts showed 100% mortality at 1000 µg/mL after 192 h [109]. Antileishmanial activity of the methanolic bark extract against *Leishmania donovani* was evaluated by in vitro antileishmanial assays, and its mechanism of action, which related to lupeol, was illustrated by in silico studies (Table 2). The results showed antipromastigote and antiamastigote activities of the extract with IC_50_ value 19.6 ± 0.9 μg/mL compared with miltefosine (3.11 ± 0.2 μg/mL) as positive control [29].

### 7.11. Antiviral Activity

Acetonic and methanolic leave extract showed over 50% reduction against HCV by infecting HCV inoculums of 3a genotype in liver cells (Huh-7 cell line) [85]. Fruit methanolic extract demonstrated inhibition of Influenza-virus-induced hemagglutination of chicken red blood cells, indicating its capability to interact with the viral hemagglutinin. In addition, it affected the nuclear transport of viral nucleoprotein. Therefore, this in vitro study suggested that *V. nilotica* can inhibit viral attachment and replication [86]. Additionally, essential oils (EOs) derived from the bark showed moderate in vitro effects against hepatitis A virus (HAV) and herpex simplex virus (anti-HSV1) in the MTT assay. This effect may be caused by its chemical constituent, caryophyllene oxide, which exhibited positive van der Waals energy interaction in silico evaluation with 3C protease of HAV and with thymidine kinase of HSV enzyme [87].

### 7.12. Antidiarrheal and Anthelmintic Activity

Methanolic bark extract proved significant antidiarrheal action against castor oil and magnesium sulfate-induced diarrhea and exhibited potent action against barium chloride-induced peristalsis of the small intestine in Swiss albino mice. Furthermore, it has in vitro antimicrobial activity against common pathogens responsible for diarrhea [59]. Similarly, in the castor oil-induced diarrhea assay performed in the same strain, *V. nilotica* methanolic roots extract at the dose of 400 mg/Kg and the standard loperamide showed inhibition of defecation by 41.37% and 58.62%, respectively [81,88], verified the in vitro and in vivo anthelmintic activities of *V. nilotica* fruits methanolic extracts against *Haemonchus contortus*. In vivo investigation on day 13 post-treatment in sheep (3 g/Kg) elucidated maximum fecal egg count reduction by 78.5%, with IC_50_ 512.9 and 195.0 μg/mL in the egg hatch test and larval development assay, respectively. These results were confirmed by another investigation on aqueous and acetone leaf extracts, which were effective against *H. contortus* and *Caenorhabditis elegans*, the highly pathogenic gastrointestinal nematode species affecting small ruminants [82].

### 7.13. Antiulcer and Healing Activities

The hydro-ethanolic extract of young seedless pods of *V. nilotica* has antiulcer activity in pylorus ligation, swimming stress and non-steroidal anti-inflammatory drugs (NSAIDs) induced ulcer rat models. The extract, containing an appreciable amount of phenolic components, possesses high antiulcer activity [110]. Clinical trials proved that the topical application of an oral paste formulation of *V. nilotica* fruits and licorice root extract alone or in combination in patients with oral ulcers could promote the healing process and reduce the diameter of the inflammatory halo of the ulcer. This paste is stable physically and chemically at room temperature and at 40 °C [55].

Daily application of vachellia pod ointment formulation for 16 days is efficient in wound healing with re-epithelization in experimental deep second-degree burns in a rat model (score 1.5, between the skin reconstruction and almost complete healing according to Kamoshida’s method) in comparison with sulfadiazine ointment which gives a similar score after 22 days of treatment [111]. *V. nilotica* methanolic leaf extract used as cream in the treatment of excision wounds made on albino rats hasten wound healing compared with the control group [91]. Application of pod extract cream for 14 days promoted wound healing in Sprague Dawley rats. The histopathological findings revealed re-epithelization, dermal tissue regeneration and angiogenesis. Besides, it significantly suppressed the expression of both TNF-α and interleukin1β (IL-1β) in the granulation tissues compared to untreated rats. These proinflammatory cytokines inhibit the formation of collagen and hydroxyproline which have a crucial role in the proliferative phase of wound healing [89]. A *V. nilotica* pod and Curcuma gel mixture exhibited good wound-healing activity in rats [90].

## 8. Toxicological Studies and Safety

*V. nilotica* has been widely used as traditional medicine in Unani and Ayurveda medicine systems for hundreds of years with no reports of toxicity or adverse effects [39]. A few studies are available on the toxicity potential of *V. nilotica* that are mostly associated with the stem bark of the plant [112]. Acute toxicity of 3 g/Kg aqueous pod extract administrated orally as a single dose produced no mortality in the treated Wister albino rats of both sexes during the 48 h after administration [113]. The aqueous stem bark extract 1 g/Kg administrated orally and intraperitoneally in mice models daily for 28 days caused no mortality and did not cause any significant histopathological lesion in the liver, brain, kidney, lung, spleen, heart or testes when compared with those of the normal control mice, but caused subclinical effects such as decreased platelets and increased creatine kinase, total bilirubin and γ-glutamyl transpeptidase levels [114]. Intraperitoneal administration of the methanol extract of stem bark to mice for 72 h revealed 50% mortality at 2 g/Kg body weight [83]. Cytotoxicity of methanolic bark extract of *V. nilotica* was tested in vitro by Alamar blue assay in human hepatoma cell line (HepG2-cells) and by calcein acetoxymethyl (Calcein-AM) uptake test in HeLa cells (human cervical carcinoma) at a concentration range from 8 to 500 μg/mL. The results showed that vachellia with a minimal dosage of 250 μg/mL has a toxic effect on mitochondrial activity by Alamar blue assay (reduced the NADPH content) and induced cellular membrane damage with Calcein-AM [97,115] reported that ethanolic leaf extract of *V. nilotica* had no hemolytic activity in vitro against rat or human erythrocytes. The toxicity of the plant not only depends on its own properties but is also clearly related to the type of solvent used, dosage rate, route and duration of consumption.

## 9. Conclusions and Future Perspectives

The present review evidences the wide traditional uses of *V. nilotica*, in relation to its main potential chemical constituents, that could account for its therapeutic properties.

The search strategy conducted for this narrative review was implemented using the principal keywords “*Acacia nilotica*” and “*Vachellia nilotica*”. The recent renaming of *Acacia nilotica* to *Vachellia nilotica* due to molecular characterization remains still controversial for many botanists and especially for certain locations, such as in Africa, where “the acacia” is iconic [116]. For search strategies, the number of recent publications referring to “acacia nilotica” instead of “vachellia nilotica” is still high. Hence, our inclusion criteria were based on the comprehensive analysis of the original research and previous reviews that afford pharmacological activities and traditional uses, regardless of the name change [117].

Widely distributed across tropical and subtropical regions, it has a wide use from agricultural purposes to many other commercial applications like gum production, dyeing or tanning. *V. nilotica* contains over 150 chemical constituents, which contribute to the studied biological activities. Applied computational techniques have predicted possible interactions of its bioactive compounds with potentially relevant therapeutic targets, which is promising.

Besides, different parts of *V. nilotica* have a long history of use in various forms (Table 3). This important ethnomedicinal role emphasizes the species’ versatility and helps to identify bioactive constituents with potential applications.

Traditional medicine preparation techniques for *V. nilotica*, such as water-based decoctions, alcoholic infusions, powdered formulations and oil-based extracts, closely align with research methods aimed at validating its therapeutic potential. Decoction and maceration, commonly employed with water as the primary solvent for oral or topical use [118], are mirrored in research practices where aqueous solvents have demonstrated superior extract yields compared to absolute organic solvents [119]. Ethanol and methanol-based extracts mimic these traditional methods and are particularly effective in isolating polar and semi-polar bioactive compounds, including flavonoids and tannins, which contribute to the plant’s pharmacological properties.

While traditional uses often emphasize the fruit, research confirms similar efficacy across various plant parts, including bark, leaves, and roots, further validating the overlap between traditional knowledge and scientific methodologies.

Considering the phytochemical composition of *V. nilotica*, most of the described constituents can be linked in different manners to mechanisms related to its traditional attributions and its studied pharmacological activity (Table 2). These constituents and the apparent safety and established traditional use highlight the potential of *V. nilotica* as a source of active molecules for the described applications and as an ingredient for phytochemical formulations directed to possible applications.

From previous research data, we can extrapolate these potential uses, as *V. nilotica* would be an effective treatment for microbial infections, diarrhea, wound and ulcer healing. The most robust evidence supports its antimicrobial activity and topical application, which would probably be of less toxic potential than systemic administration. There is less evidence for its uses in the treatment of diabetes, hyperlipidemia, hypertension, asthma, fever and arthritis. It should be mentioned that most mechanisms of action and the metabolic pathways of *V. nilotica* are still unclear, and some are merely the authors’ hypotheses. The implications of this knowledge gap are important, as a lack of understanding of how it exerts its effects could be an obstacle to the development of new effective and safety therapeutic compounds. We noted that some research did not include sufficient data about methodology, which limits reproducibility. This constitutes a clear limitation and implies a potential sour for research bias. Human clinical trials on *V. nilotica* are scarce and limited by small sample size and short duration. Therefore, further clinical trials should be performed to confirm its efficacy and potency in the treatment of different microbial infections, and enhanced wound healing. Despite lacking human clinical trials, the oral (infusion, decoction and in diet) and topical (mouthwash, toothpaste, ointment and cream) forms have been applied safely in humans at certain doses. More pharmacological and toxicological studies on *V. nilotica* are still necessary before recommending its formulation as therapy for human and animal diseases.

Finally, this review encompasses a wide range of pharmacological activities, an extensive coverage that provides an important understanding of vachellia’s medicinal potential. The diversity of studies, including in silico, in vitro, in vivo and clinical trials, strengthens the reliability of the findings and highlights the practical applications and potential therapeutic benefits of *V. nilotica* in human health, making this review relevant for medical and clinical research.

The growing body of scientific evidence supporting *V. nilotica*’s medicinal properties suggests its potential as a natural alternative to conventional pharmaceuticals in treating various health conditions. While prior studies largely focus on isolated pharmacological activities, such as antimicrobial, antioxidant or anti-inflammatory effects, exploring the full therapeutic spectrum synthesizes this scattered information to highlight emerging and underexplored applications.

## Figures and Tables

**Table 1:** Chemical constituents of *Vachellia nilotica* by the different parts of the plant.

Part of the Plant	Classification	Compound	References
Seeds	Tannins	Gallic acid, methyl gallate and digallic acid	[18,19]
	Flavonoids	Leucocyanidin, epicatechin, quercetin, naringenin, kaempferol and isorhamnetin	[20]
	Amino acids	Lysine, cysteine, methionine, threonine and tryptophan, leucine, histidine, valine, aspartic acid, glutamic acid, tyrosine, glycine, alanine, phenylalanine and arginine	[8,19]
	Fatty acids	Palmitic, oleic, linoleic, stearic, arachidonic and coronaric acids	[20]
Pods	Tannins	Gallic, digallic and ellagic acids	[21,22]
	Terpenes	Niloticane	[22]
	Flavonoids	Rutin and epicatechin	[21]
Leaves	Tannins	Ethylgallate	[23]
	Flavonoids	Rutin, flavone and querstin 3-galactosyl	[24]
	Terpenes	Lupeol	[25]
Bark	Tannins	Gallic acid, epigallocatechin-5,7-digallate and dicatechin	[26]
	Flavonoids	Catechin, Kaempferol, Leucocyanadin, Acacetin and Rutin	[27]
	Terpenes	Betulin, lupeol and lupenone Niloticane	[28,29,30]
Flower	Tannins	Gallic acid	[31]
	Flavonoids	Quercetin, quercetin 3-O-β-glucoside, catechin, catechin 7 O-gallate, naringenin and naringenin 7-O-β-glucopyranoside	

**Table 2:** Computational approaches of reported *Vachellia nilotica’s* phytochemical constituents.

Pharmacological Activity	Phytochemical Constituents	Target of the Interaction	Consequences	Method	Reference
Anti-diabetic	Ellagic acid	Insulin receptor tyrosine kinase	Increase in glucose uptake, lowering blood glucose levels	Molecular docking and Molecular Mechanics/Poisson–Boltzmann Surface Area (MM/PBSA) energy calculations	[11]
	Rutin	α-amylase and α-glucosidase enzymes	Inhibition of carbohydrate digestion, lowering postprandial blood glucose levels	Molecular docking, molecular dynamics simulation and MM/PBSA	[33]
	Quercetin Gallic acid	Glycogen phosphorylase enzyme	Glycogenolysis inhibition, reducing hyperglycemia	Molecular docking	[34,35]
		Peroxisome proliferator-activated receptor gamma	Increased insulin sensitivity	Molecular modeling, molecular docking and molecular dynamics simulation	
	Rutin	Sodium-glucose co-transporter 2 (SGLT-2)	Inhibition of renal glucose reabsorption, leading to a lowering in plasma glucose level	ADMET profiling, molecular docking, molecular dynamics simulations and MM/PPBSA energy calculations	[36]
	Quercetin Rutin	Aldose reductase enzyme	Limiting the development of diabetic complications by inhibition of hyperglycemia-induced polyol pathway	Molecular docking, molecular dynamics simulation and MM/PBSA	[33,37]
Anti-inflammatory	Quercetin	Phospholipase A2 enzyme prostaglandin G1 /H1 synthase prostaglandin G2 /H2 synthase	Prevents the inflammatory response by reducing inflammatory mediator synthesis through the inhibition of arachidonic acid	Molecular docking	[38]
	Quercetin Kaempferol Ellagic acid	Xanthine oxidase	Prevents formation of uric acid (triggers nonspecific inflammation response) and superoxide radicals	Molecular docking, molecular dynamics simulation and MM/PBSA	[37,39]
	Quercetin Ellagic acid	Cyclooxygenase-2 (COX-2) Lipoxygenase- 5 (LOX-5)	Prostaglandin and leukotriene biosynthesis inhibition	Molecular docking	[40]
Antidiarrheal	Quercetin	Mu and delta opioid receptors	Central inhibition of diarrhea	ADMET profiling, molecular docking and molecular dynamics simulations	[32]
Antimicrobial	Gallic acid	Aquaporin	Antibacterial effect by disrupting cell membrane integrity and affecting bacterial viability	Molecular docking	[41]
		Telomerase enzyme	Anti-proliferative effect by inducing deterioration in the enzyme structure		
	Rutin Quercetin Kaempferol	Trehalose-6-phosphate phosphatase (TPP)	Inhibition of this enzyme deprives the organisms (Mycobacterium, Aspergillus and some nematodes) of trehalose biosynthesis	Molecular docking	[42]
	Quercetin	Heat shock protein	Cytoprotective effect and regulation of immune response	Molecular docking	[43]
		Surfactant protein	Inhibition of the growth of gram-negative bacteria by increasing membrane permeability		
		Lactobacillus bacterial protein	This interaction has a role in septic urinary infection therapy		
	Lupeol	Leishmanial enzymes: ▪ Trypanothione reductase; ▪ Adenine phosphoribosyl transferase; ▪ Sterol 24-c-methyltransferase; ▪ Pteridine reductase.	Antileishmanial effect	Molecular modeling, molecular docking and molecular dynamics simulation	[29]
	Gallic acid	HIV-1 protease	Suppresses viral replication	Molecular docking	[44]
	Quercetin	Hepatitis C virus (HCV) Non-structural protein 5A Influenza A virus (IAV) nucleoprotein (NP) specific inhibitor	Interferes with viral replication	Molecular docking	[45]
	Quercetin Gallic acid	SARS-CoV-2 main protease (M^pro^)	Interferes with viral replication and transcription	Molecular modeling, molecular docking, molecular dynamics simulation	[46]
	Kaempferol Gallic acid	SARS-CoV-2 RNA-dependent RNA Polymerase (RdRp)			
Anticancer activity	Quercetin	DNA topoisomerase I enzyme	Disrupts DNA structures and delays replication	Molecular modeling and docking; rescoring procedure and hydropathic analysis	[47]
	Quercetin Catechin	Tyrosine-protein kinase Lyn	Stops cancer cells from growing and dividing	GRID and docking analyses	[48]
	Quercetin Naringenin	Aromatase enzyme and estrogen receptor beta.	Anti-breast cancer activity by modulation of estrogen signaling	Molecular modeling	[49]
Neuromodulatory activity	Rutin Epicatechin	Acetylcholinesterase and Butyrylcholinesterase enzymes	Promotes signaling amongst nerve endings and enhances their potential in the cholinergic pathways	ADMET profiling and molecular docking	[50]
		Monoamine oxidases enzyme	Increases synaptic levels of dopamine, serotonin and norepinephrine		
Antiplatelets activity	Quercetin	The P2Y12 receptor (G-inhibitory-protein receptor on the platelet membrane)	Inhibition of platelet activation, management and prevention of arterial thrombosis	ADMET profiling and molecular docking	[51]

**Table 3:** Ethnomedicinal uses and preparation forms of different parts of *Vachellia nilotica*.

Plant Parts	Preparation Forms	Ethnomedicinal Uses	References
Pods	Decoction	Dry cough, urinary tract infections and cases of increased urine frequency. Albuminuria, glucosuria, urine turbidity and urogenital disorders.	[52,53]
	Powders	Management of blood glucose levels.	[54]
	Paste	Oral ulcer.	[55]
	Vaginal pessary	Abnormal vaginal discharge and associated symptoms.	[56]
Leaves	Infusion	On wounds to stop bleeding, anti-inflammatory, astringent for diarrhea, dysentery, acute leucorrhea, gonorrhea, as a liver tonic, strengthen vision, eye diseases, as a gargle to cure sore throat, spongy gums, and also as a wash in hemorrhagic ulcers and wounds.	[57]
	Decoction	Gastrointestinal tract and eye diseases, bronchitis and fractures healing.	
	Paste	Itching.	
Root	Infusion	Tuberculosis, bronchitis, asthma, gastro-enteritis, diarrhea, anorexia, appetite enhancer, nutrient supplement, stomachache, indurations of liver and spleen, cancer, tumors, painful joints, tinnitus, dental care and cleaning circumcision wounds.	[58]
Bark	Decoctions	Treatment of diarrhea, dysentery and liver disorders. Improve digestion, mouth ulcers, toothache, bronchitis, sore throat, dry cough, asthma, children’s fevers, cystitis, vaginitis and as a nerve stimulant.	[30,59]
	Juice	As a dropper for conjunctivitis (mixed with breast milk).	[60]
	Toothpaste	Dental caries.	
Gum	Gel	Useful in plaque and gingival conditions.	[61]
	Pessary	Uterine prolapse.	[62]
	Powder	Mixed with quinine for fever complicated with diarrhea and dysentery. Mixed with egg white applied on burns and scalds. An emollient, liver tonic, antipyretic and antiasthmatic.	[53,63]

**Table 4:** Summary of pharmacological activities of *Vachellia nilotica*.

Pharmacological Activity	Study	Outcome	Part Used	Extract	References
Glucose and lipid-lowering	In vivo	Improvement of these values in animal models compared to the diabetic control group: blood glucose level, plasma insulin and C-peptide levels, HbA1c, cholesterol, triglyceride, LDLC, HDLC and VLDLC	Leaves	Polyphenolic extract (250–500 mg/kg) in alloxanized rats. Aqueous extract (300 mg/Kg) in STZ diabetic rats	[64,65]
				Ethanolic extract (30 mg/150 g/day, I.P., for 7 days and 4 weeks) in (AIHRs) model	[66]
			Bark	Ethanolic extract (250 mg/kg for 21 days) in STZ diabetic rats	[67]
				Aqueous methanolic extract	[68]
			Pods	Ethanolic extract (200 mg/kg) in fructose-induced hyperlipidemic rats	[69]
				Hot water extract	[70]
				Aqueous methanolic extract	[71,72]
		Attenuating hyperglycemia in T1D alloxanized mice; lowering insulin resistance and systemic glucose uptake; attenuating diabetes complications such as dyslipidemia, hepatic injury and nephrotoxicity	Leaves	Ethanolic extract (200 mg/Kg/day, orally, for 20 days) in alloxanized mice	[73]
	In vitro	α-Glucosidase inhibitory and pancreatic lipase inhibitory activities	Pods	Ethanolic extract Water extract	[74]
		α-Glucosidase inhibition by 98% vs. 56% for acarbose at 100 µg/mL of both, IC_50_ value of the extract 8 µg/mL	Bark	Ethanolic extract	[67]
		Lipogenic Activity (The murine 3T3-L1 embryonic cell line) 1.70 vs. troglitazone 1.43	Bark	Aqueous methanolic extract (50 µg/mL)	[68]
Antioxidant activity	In vivo	Improvement in hepatic and pancreatic antioxidant defense markers levels (GSH, SOD, CAT, GPx); reduction in hepatic and pancreatic ROS and TBARS levels	Leaves	Polyphenolic extract (250 mg/kg and 500 mg/kg) in alloxanized diabetic rats	[64]
	In vitro	Potent antioxidant activity (DPPH radical scavenging assay), IC_50_ value 4.06 ± 0.09 and 7.51 ± 0.19 μg/mL for ethanol and water extracts vs. Trolox of 11.35 ± 0.05 μg/mL	Pods	Ethanolic extract Water extract	[74]
		Significant radical scavenging activity in different in vitro assays: DPPH scavenging assay; deoxyribose degradation assay; chelating effects on ferrous ions; reducing power assay; lipid peroxidation by thiobarbituric acid (TBA) assay	Bark	Kaempferol (polyphenolic compound from methanol extract)	[27]
		Hydrogen peroxide free radical and DPPH scavenging assay	Stem bark	Petroleum ether, ethyl acetate and methanol extracts	[75]
Anti-inflammatory activity	In vivo	Marked anti-inflammatory activity against formalin-induced paw edema in albino mice by reduction of paw diameter to 57.16% vs. Diclofenac 56.30%	Leaves	Aqueous extract (150 mg/kg)	[76]
		Reduction in carrageenan-induced paw edema in rats 20% vs. 47% aspirin (100 mg/Kg)	Pods	Aqueous extract (500 mg/Kg)	[77]
		Inhibit rat paw edema induced by carrageenan to 64.41% vs. 65.11% indomethacin (10 mg/Kg); inhibit rat granuloma formation induced by the cotton pellets 25.62% vs. 37.64% dexamethasone (2.5 mg/Kg)		Aqueous extract (100 mg/Kg)	[78]
	Clinical trial (randomized, placebo and standard controlled)	Significant reduction in gingival and plaque index scores compared to a placebo gel control group without teeth discoloration or unpleasant taste	-	Commercial vachellia gel in patients with chronic generalized gingivitis	[63]
	In vitro	Inhibition of TNFα-stimulated 3T3-L1 adipocytes (The murine 3T3-L1 embryonic cell line) 50% vs. troglitazone 29% (5 µg/mL)	Bark	Aqueous methanolic extract (50 µg/mL)	[68]
Antinociceptive activity	In vivo	Significant analgesic effect for both acute (50 mg/Kg) and chronic pain (100 mg/Kg) estimated by formalin-induced writhing test on albino mice and compared with the untreated mice. The findings suggest both direct analgesic effects on the nociceptor blockage and an inhibition of the synthesis and/or release of inflammatory pain mediators.	Bark	Aqueous extract (50, 100 mg/Kg)	[76]
		Significant increase in reaction time assessed by the hot plate test in mice with a maximum of 90 min vs. 30 min aspirin (100 mg/Kg)	Pods	Aqueous extract (500 mg/Kg)	[77]
Antipyretic activity	In vivo	Antipyretic activity against brewer’s yeast-induced pyrexia in albino mice 98.23% compared to paracetamol 99.03%	Bark	Aqueous extract (150 mg/kg)	[76]
		Inhibitory effect on yeast-induced pyrexia in rats	Pods	Aqueous extract (500 mg/Kg)	[77]
Antibacterial activity	In vitro	Inhibitory activity on disc diffusion assay against gram-positive bacteria (*Bacillus*, *Staphylococcus aureus*) and gram-negative bacteria (*Salmonella*, *Shigella*, *Vibrio*, *Pseudomonas*, *Klebsiella* and *E. coli*); the zone of inhibition around 9–13 mm vs. Ciprofloxacin (10 µg/mL) 33–44 mm	Stem bark	Petroleum ether and ethyl acetate extracts (300 µg/mL)	[75]
		Antibacterial activities against *Pseudomonas flurorescens*, *Bacillus subtilis* and *E. coli* by agar well diffusion assay; the zone of inhibition is around 6–22 mm vs. Ciprofloxacin 22 mm		Different ratio of methanolic chloroform extracts (25:75), (50:50) and (75:25)	[79]
	Clinical trial (single-blind, randomized controlled)	*V. nilotica* has similar effects as metronidazole against bacterial vaginosis	Bark	Decoction of 30 gm twice daily to 30 patients orally for 1 month and metronidazole (400 mg twice daily) for 7 days to 15 women as control	[80]
Antifungal activity	In vitro	Inhibitory activity on disc diffusion assay against *Candida*, *albicans*, *Candida arrizae*, *Candida krusei*, *Aspergillus fumigatus*, *Aspergillus niger*, *Rhizopus oryzae* and *Saccharomyces cerevisiae*; the zone of inhibition is around 7–8 mm vs. griseofulvin (25 µg/mL) 14–24 mm	Stem bark	Petroleum ether and ethyl acetate extracts (300 µg/mL)	[75]
		Antifungal activities against; *Aspergillus niger*, *Fusarium oxysporium* and *Dreschlera avenacea* by agar well diffusion assay; the zone of inhibition is around 6–10 mm vs. ketoconazole 20 mm		Different ratios of methanolic chloroform extracts (25:75), (50:50) and (75:25)	[79]
Anthelmintic activity	In vitro	Anthelmintic activity against *H. contortus* by these assays: the adult motility; the egg hatch; and the larval development	Fruits	Methanol extract	[81]
	In vivo	Treatment of sheep naturally infected with *H. contortus* for 13 days lessened the fecal egg by (78.5%)			
		Effective against *H. contortus*- and *C. elegans*-affected sheep	Leaves	Aqueous and acetone extracts	[82]
Antiprotozoal activity	In vivo	Trypanocidal activity against *Trypanosoma brucei* infection in mice; the partially purified extract of 50 mg/kg cleared parasites from circulation within 2 days, and the crude extract, 400 mg/kg, within 8 days	Stem bark	Methanol extract (crude and partially purified extract)	[83]
	In vitro	The susceptibility assays against *Giardia lamblia* trophozoites showed 100% inhibition by the extract (500 µg/mL) after 96 hrs. vs. metronidazole 96% inhibition at concentration 312.5 µg/mL at the same time	Leaves	Ethanolic extract	[84]
		Antileishmanial activity against *Leishmania donovani* by antileishmanial assays showed antipromastigote and antiamastigote activities of the extract with IC_50_ value 19.6 ± 0.9037 μg/mL vs. miltefosine (3.118 ± 0.2395 μg/mL) as positive control	Bark	Methanolic extract	[29]
Antiviral activity	In vitro	Showed over 50% reduction against HCV by infecting HCV inoculums of 3a genotype in liver cells (the Huh-7 cell line)	Leaves	Acetone and methanolic extract	[85]
		Inhibition of Influenza virus-induced hemagglutination of chicken red blood cells	Fruits	Methanolic extract	[86]
		Using MTT assay, essential oils showed in vitro anti-hepatitis A virus and anti-herpex simplex virus-1	Bark		[87]
Antidiarrheal	In vivo	Significant antidiarrheal action against castor oil and magnesium sulfate-induced diarrhea in the Swiss albino mice model	Bark	Methanolic extract	[59] [88]
			Root		
		Significant antidiarrheal action against barium chloride-induced peristalsis of small intestine in mice	Bark		[59]
Wound healing	In vivo	Promoted wound healing through antioxidant properties and suppressing proinflammatory cytokines in SD rats	Pods	Aqueous extract	[89]
		Mixture formulation with Curcuma gel exhibited significant wound-healing activity in rats		Powder incorporated into gel medium	[90]
		Hasten wound healing than the control group when used as cream in the treatment of excision wounds made on albino rats	Leaves	Methanolic extract	[91]
Antihypertensive and antispasmodic	In vivo	Lowering arterial blood pressure in rats	Pods	Methanolic extract	[92]
	In vitro	Inhibited the rate and force of spontaneous contractions in guinea-pig atria and rabbit jejunum.			
Antiplatelet aggregatory activity	In vitro	On inducible human platelets aggregation, exhibited antiplatelet aggregatory activity due to Ca^2+^ channels blockade and protein kinase C effect	Fruits	Methanolic extract	[93]
	In vivo	Significant antiplatelet aggregation (4.35%) compared with normal and diabetic rats (2.11% and 8.76%, respectively) in STZ-induced diabetic rats	Leaves	Methanol extract (50 mg/Kg for 3 weeks)	[65]
